# An Ounce of Prevention: Reducing Ageism Through the Lens of Public Health

**DOI:** 10.1093/geroni/igab046.2790

**Published:** 2021-12-17

**Authors:** Erin Grinshteyn

**Affiliations:** University of San Francisco, San Francisco, California, United States

## Abstract

Ageism is pervasive. The negative consequences of ageism are vast, and the literature on the effects of ageism on health and health care is extensive. The perpetrators of ageism are equally vast. While it may be tempting to believe that those who go into the fields of gerontology and geriatrics are free from these attitudes and behaviors, this is untrue. It is reasonable to suspect that future public health professionals, even those interested in gerontology, may also carry ageist ideas and practices into their professional careers. This research was developed to determine whether teaching about aging and ageism in a public health course could reduce ageism among students. Participants were students in a class on aging and public health. All students were graduate students in a Master of Public Health (MPH) program. Multiple assessments were used to assess ageism including the Framboni Scale of Ageism (FSA), a validated 29-question measure used to assess ageism, and the Succession, Identity, and Consumption (SIC) scale, another scale assessing ageism. Students were enrolled in an elective course on aging and public health, which was taught through the public health lens of disease prevention and health promotion. Health topics related to aging are discussed with an emphasis on prevention. The contributions older adults make, and the resulting improved health and well-being of self, others, and community are promoted. And the class participates in activities with a variety of community-dwelling older adults. Results show that ageism among students is reduced after the semester long course.

